# Social Media Use Among Living Kidney Donors and Recipients: Survey on Current Practice and Potential

**DOI:** 10.2196/jmir.6176

**Published:** 2016-12-20

**Authors:** Abby Swanson Kazley, Bashir Hamidi, Wendy Balliet, Prabhakar Baliga

**Affiliations:** ^1^ Department of Health Care Leadership and Management Medical University of South Carolina Charleston, SC United States; ^2^ Department of Surgery Medical University of South Carolina Charleston, SC United States; ^3^ Department of Psychiatry and Behavioral Medicine Medical University of South Carolina Charleston, SC United States

**Keywords:** living kidney donation, kidney transplant, social media

## Abstract

**Background:**

In the United States, there is a national shortage of organs donated for transplant. Among the solid organs, most often kidneys are donated by living donors, but the lack of information and complicated processes limit the number of individuals who serve as living kidney donors. Social media can be a tool for advocacy, educating the public about the need, process, and outcomes of live kidney donors, yet little is known about social media use by kidney transplant patients.

**Objective:**

The purpose of this study was to examine the social media use of potential kidney transplant patients and their willingness to use social media and their networks to advocate and educate about living kidney donation.

**Methods:**

Using a validated survey, we modified the instrument to apply to the patient population of interest attending the Medical University of South Carolina, Charleston, SC, USA. The questions on the survey inquired about current social media use, sites visited, frequency and duration of social media use, and willingness to use social media to share the need for living kidney donors. We asked patients who had received a transplant and those awaiting a transplant to complete the survey during an office visit. Participation was voluntary.

**Results:**

A total of 199 patients completed the survey. Approximately half of all kidney transplant patients surveyed used social media (104/199, 52.3%), and approximately one-third (66/199, 33.2%) had more than 100 friends in their social media network. Facebook was the most popular site, and 51% (102/199) reported that they would be willing to post information about living kidney donation on their social networks. More than a quarter of the sample (75/199, 37.7%) had posted about their health status in the past.

**Conclusions:**

Social media holds great promise for health-related education and awareness. Our study shows the current social media use of kidney transplant patients. In turn, such information can be used to design interventions to ensure appropriate decision making about live kidney donation. Transplant programs can help increase the number of living donors by providing guidance to kidney transplant patients in how to use social media, to be advocates, and to provide information about living kidney donation to their social network.

## Introduction

For patients with end-stage renal disease, kidney transplant is the best available treatment, improving longevity of life (average 10 years), optimizing quality of life, and costing significantly less than dialysis treatments. One of the biggest challenges in the US transplantation community (though this problem exists worldwide) is the growing gap between the increasing demand and the number of procured organs [1,2]. In the United States, there are more than 100,000 candidates on the kidney transplant waitlist. However, only 17,878 kidney transplants were performed in the United States in 2015 [3].

Recent strategies to reduce waitlist time-to-transplant have focused on increasing living donor kidney transplant (LDKT) [4-6]. LDKT is associated with improved graft function, better patient survival, and lower health-related financial costs [7]. Although the risks of donation are minimal (eg, potential hypertension, hernia, and <0.01% mortality), there has been an overall decrease in rates of LDKT [4,8]. Barriers to living kidney donation include difficulty communicating about the need for kidney donation with potential donors, worry about financial strain for the donor, fear of health risks to the donor, and a general lack of knowledge about the kidney donation and transplant process. Patients and transplantation professionals must continue to seek new ways to promote awareness and provide education about the opportunity to be a living donor.

Social media has proven to be an effective tool for increasing awareness about various chronic illnesses and has been used to promote positive health behavior change for certain health-related topics. The literature supports the utility of social media campaigns to promote awareness about cancer and to reduce rates of smoking, alcohol abuse, and use of illicit drugs [9-11]. Further, social media has been shown to reinforce positive behaviors such as healthy eating, regular exercise, and frequent preventive medical screenings [12]. One of the notable campaigns using social media, with a goal of increasing the number of donor registrations, has been a remarkable collaborative effort between the transplantation team at the Johns Hopkins University School of Medicine and Facebook that resulted in the addition of a feature on Facebook that both raised awareness about donation and recruited participants into donor registries. Known as the “Facebook effect,” this effort was vastly successful, increasing the number of new donor registrations by approximately 21-fold by the day after the implementation and by a 5.8-fold increase over baseline over a 13-day period [13].

These innovative health-promoting social media initiatives highlight the great potential to increase awareness and provide education about living kidney donation by capitalizing on the influence of social media in the United States. Social media can be a tool used in creative ways to educate hard-to-reach populations. With the worldwide use of social media and its structure of social group communication, there can be opportunities for patients to use their social media networks as a way to increase LDKT, with the goals of reducing time on the waitlist and improving quality of life both before and after transplant. This can be accomplished by raising awareness and educating others about the need for living kidney donation. However, the literature is lacking in understanding whether or how potential transplant patients use social media. The purpose of this study was to examine and describe social media use by individuals in South Carolina who have been referred for a kidney transplant. This study allowed us to learn about patients’ current practices with social media and evaluate the potential for future interventions that could raise awareness of the opportunity for living kidney donation through the use of social media.

## Methods

We used a cross-sectional design to examine patients’ current use of social media for health-related and nonhealth-related topics. Additionally, we assessed patients’ perceptions of social media use for specific kidney transplant health-related activities. Study data were collected and managed using REDCap electronic data capture tools hosted at the Medical University of South Carolina, Charleston, SC, USA [14]. A research associate administered approximately 200 survey interviews to both kidney transplant candidates and kidney transplant recipients who were seen at the Medical University of South Carolina Renal Access Clinic. Kidney transplant candidates were defined as patients who did not have a kidney transplant prior to administration of the survey and who were attending clinic as a pretransplant requirement to be placed on our kidney transplant waitlist. Kidney transplant recipients were defined as those who underwent a kidney transplant and were attending clinic visits as a routine follow-up or due to an acute issue.

Data collection was not identified to any particular patients and data were only examined in the aggregate. Patients provided verbal consent during their clinic visit and, if they were willing to participate, were interviewed using the survey questions. Data were collected between May and September 2015, and the study was approved by the institutional review board of the Medical University of South Carolina.

Using a validated survey with permission from PricewaterhouseCoopers LLP (New York, NY, USA), we sought to identify social media use in our sample [15]. Due to the specific aims of our study, we added questions to the survey to gauge the willingness of patients to share transplant-related information. The added questions were written with expert input and pilot tested with a sample of 15. Descriptive analyses, including frequencies, means, and ranges, were calculated for all the variables. We computed 1-way analysis of variance and chi-square analyses to determine any potential differences among transplant status, and social media use and demographic variables (eg, age, income, education, sex), using IBM SPSS version 23 (IBM Corporation).

## Results

The total number of patients surveyed was 199. Of these 199 patients, a majority (n=115, 57.8%) were male. A majority of the patients were kidney transplant recipients (n=133), and 66 were kidney transplant candidates. In terms of educational level attained, 29 patients had not graduated from high school, and 8 had an eighth grade education or less. More than one-third had graduated from high school, and more than half had at least some college education. The majority of the sample ranged in age from 45 to 64 years, while 21.1% (n=42) were older than 65 years, and 24.1% (n=48) were less than 45 years of age. Table 1 lists these results.

More than half of the sample (n=104, 52.3%) reported that they used social media sites on the Internet. Due to small sample sizes within groups, we transformed age to include only 2 groups (18-44 and ≥44 years), and we collapsed education to comprise 3 groups (less than a high school diploma, high school degree and some college, 2-year college degree or higher). Pearson chi-square analyses comparing potential differences between transplant status (pre- or post-) and social media use revealed no significant difference between those who had a transplant and those who had not in their tendency to use social media (χ^2^_12_=.023, *P*>.99) *.* No significant differences were found between transplant status and income, sex, age, or education. In terms of social media use, there was a significant difference between age and social media use, with younger people more likely than older adults to use social media (χ^2^_8_=26.15, *P*<.001). We found no significant differences between social media use and any other demographic variables.

Nearly half (n=96) of the patients sampled had been using social media for less than a month, whereas 31.2% (n=62) had been on social media for over 4 years. Of those who used social media, 24.1% (n=48) reported using social media for 5 hours or less per week. Given the purpose of the study, the size of the social media networks was of particular interest. Of those on social media, 33.2% (n=66) had over 100 “friends” in their social network. Table 2 presents these results.

The most frequently used social media site was Facebook, followed by Instagram, Twitter, and Google+. None of the participants used Reddit, Foursquare, or LiveJournal. Table 3 presents these results.

**Table 1:** Descriptive data of kidney transplant candidates and kidney transplant recipients, Medical University of South Carolina Renal Access Clinic, May-September 2015 (N=199).

Characteristics		n (%)
**Sex**		
	Male	115 (57.8)
	Female	84 (42.2)
**Transplant status**		
	Pretransplant	66 (33.2)
	Posttransplant	133 (66.8)
**Age range (years)**		
	18-24	5 (2.5)
	25-34	14 (7.0)
	35-44	29 (14.6)
	45-54	54 (27.1)
	55-64	55 (27.6)
	≥65 years	42 (21.1)
**Educational level attained**		
	≤8th grade	8 (4.0)
	Some high school	21 (10.6)
	Graduated from high school	69 (34.7)
	Some college	41 (20.6)
	2-year college	25 (12.6)
	≥4-year college	35 (17.6)

**Table 2:** Summary of social media use by kidney transplant candidates and kidney transplant recipients.

Survey questions		n (%)
**Do you use social networking sites on the Internet?**		
	Yes	104 (52.3)
	No	95 (47.7)
**How long have you been on social media?**		
	<1 month	96 (48.2)
	1-6 months	3 (1.5)
	6-12 months	9 (4.5)
	1-2 years	10 (5.0)
	2-4 years	18 (9.0)
	≥5 years	62 (31.2)
	Missing	1 (0.5)
	Total	199
**How many hours per week do you spend on social media?**		
	N/A^a^	97 (48.7)
	0-5 hours	48 (24.1)
	6-10 hours	29 (14.6)
	11-20 hours	10 (5.0)
	21-30 hours	5 (2.5)
	≥31 hours	9 (4.5)
	Missing	1 (0.5)
	Total	199
**How many “friends” do you have on social media?**		
	0-10	6 (3.0)
	11-50	17 (8.5)
	51-100	15 (7.5)
	101-250	22 (11.1)
	≥251	44 (22.1)
	Total	104
	N/A	95

^a^N/A: not applicable.

**Table 3:** Social media of choice among kidney transplant candidates and kidney transplant recipient.

Social media site used	n
Facebook	102
Google+	16
Tumblr	2
Twitter	18
Instagram	20
Pinterest	13
Reddit	0
Vine	3
LiveJournal	0
Foursquare	0
LinkedIn	12
Myspace	2
Snapchat	8
Other	3

Once we established that the majority of our sample used social media, we sought to gauge their comfort and willingness to use it to promote health-related causes. Results indicated that 25.1% (n=50) of patients questioned had used social media to post health-related activities, while 37.7% (n=75) had posted about nonhealth-related activities. Importantly, 35.7% of participants (n=71) reported that they would be willing to share information about their health through social media. And nearly half (n=94, 47.2%) of patients indicated that they would be willing to post information about their kidney disease on social media. Survey results revealed that several patients or their families had already used social media to promote their need for living kidney donation: 14 patients (7%) had a family or friend post about their need for a living kidney donor, 3 patients had asked for a living kidney donation through social media, and 6 (3.0%) had both posted themselves and had a friend or relative post for them. In total, 23 individuals (11.6%) had used social medial to ask for a living kidney donation. Table 4 presents these results.

**Table 4:** Health-related social media use and willingness to use it to promote health-related causes.

Survey questions		n (%)	
**Have you posted health-related activities using social media?**		
	Yes	50 (25.1)
	No	54 (27.1)
	Total	104
	N/A^a^	95 (47.7)
**Have you posted nonhealth-related activities on social media?**		
	Yes	75 (37.7)
	No	28 (14.1)
	Total	103
	N/A	96 (48.2)
**Would you be willing to share information about your health through social media?**		
	Yes	71 (35.7)
	No	33 (16.6)
	Total	104
	N/A	95 (47.7)
**Would you be willing to post information about your kidney disease on social media?**		
	Yes	94 (47.2)
	No	41 (20.6)
	Total	135 (67.8)^b^
	Missing	64 (32.2)
**Did you or a family or friend share information on social media about your need for a kidney transplant?**		
	Neither asked	74 (37.2)
	Patient asked	3 (1.5)
	Family or friend asked	14 (7.0)
	Both asked	6 (3.0)
	Total	97 (48.7)
	Missing	102 (51.3)

^a^N/A: not applicable.

^b^Includes people who said “no” to a history of sharing.

## Discussion

The utility of social media holds great potential for connecting individuals with others, as well as for providing health-related education. Results from this study revealed that social media is used by many patients in this population who are in need of, or already had, a kidney transplant. Importantly, responses indicate that almost half of patients surveyed would be willing to use social media to share information about their kidney disease. Younger adults are more likely to use social media, which suggests that older adults (≥45 years) could be targeted as a population of intervention to educate about social media use. The results demonstrate the vast potential that social media has for raising awareness about chronic illness and providing health care-related education.

From a transplant-specific perspective, we have shown that some patients and family members have already begun using social media to promote the need for living kidney donors. Moreover, many patients who haven’t yet used social media in this way indicated a willingness to do so. Because social media has been effective in raising awareness for other health-related topics, the global potential in using it as a tool to raise awareness about renal disease and the prospect of living donor transplants is immeasurable. One of the significant barriers to living kidney donation is the kidney transplant candidate’s hesitation in initiating conversation with potential donors, family, and friends. Therefore, social media use offers an innovative and unique opportunity for transplant candidates to promote awareness about renal disease and the negative effect of various treatments (eg, dialysis), and about their need for a kidney, in a thoughtful and less anxiety-provoking way, thereby potentially increasing their network size of potential living kidney donors. Despite the sensitivity and privacy that sometimes accompany illness, results from this study suggest that a good number of patients would be willing to share information about their kidney disease using social media, yet only a small number have done so.

Interestingly, kidney disease patients sampled in this study used social media less than the general population of adults in the United States (65%) [16]. Results then also suggest that the kidney transplant population is actually underrepresented on social media. This indicates that patients may need guidance in using social media in general, and also to promote their need for a transplant. It is also possible that many of the patients who do use social media have not yet considered using it for this purpose.

Given the potential networking capability of social media, a well-presented case of an individual patient in need of a kidney transplant may raise awareness and lead individuals who know the patient, as well as altruistic donors, to come forward. Since Gallup data have shown that a majority of Americans support organ donation, there is reason to believe that individuals would be receptive to such targeted messages [17]. There have been a handful of already publicized cases of living donors who came forward after learning of the need of an individual patient through social media [18,19]. Furthermore, with the option of paired kidney exchanges and donation chains, the potential for donations to happen is even greater than if a single person comes forward to make a single donation. Given the abundant and growing need for transplantable organs, social media may provide a tool to increase awareness and promote growth in living kidney donation.

Future research may evaluate the use of a social media intervention to increase public awareness of the potential to serve as a living kidney donor and, subsequently, the number of living donors who are evaluated and have learned of the option of living kidney donation through a social media app. Such interventions will have to heed which social media sites are the most popular, as the goal would be to reach the widest audience possible. Transplant centers may consider providing coaching or examples of how social media messages should be written to be compelling, accurate, and informative. Although some patients have larger social media networks (ie, number of friends), others do not. Those who do not can still use the tool by asking their “connections” to also share their message, with the option to boost posts geographically.

Our study does have limitations. First, the data are from only a single transplant center and, thus, the results may not be representative of other geographical areas. Second, the results are self-reported and thus may include some responder bias. It was surprising that 48% of participants had only used social media for less than a month; this may suggest survey reading confusion. We also did not gather information about etiology of kidney disease or ethnicity, which could have added to our understanding of social media use in this population. Third, this was an exploratory study examining the potential of social media in living kidney transplant, and our results are not tied to any specific outcomes.

## Abbreviations

LDKT: living donor kidney transplant
